# Comparative transcriptome analysis reveals candidate genes for cold stress response and early flowering in pineapple

**DOI:** 10.1038/s41598-023-45722-y

**Published:** 2023-11-02

**Authors:** Ashley G. Yow, Kanjana Laosuntisuk, Roberto A. Young, Colleen J. Doherty, Nicholas Gillitt, Penelope Perkins-Veazie, Qiu-Yun Jenny Xiang, Massimo Iorizzo

**Affiliations:** 1 Department of Horticultural Science, North Carolina State University, Raleigh, NC 27695 USA; 2 Plants for Human Health Institute, North Carolina State University, Kannapolis, 28081 USA; 3 Research Department of Dole, Standard Fruit de Honduras, Zona Mazapan, 31101 La Ceiba, Honduras; 4 Department of Molecular and Structural Biochemistry, North Carolina State University, Raleigh, NC 27695 USA; 5 Berkley LLC, Kannapolis, NC 28081 USA; 6 Department of Plant and Microbial Biology, North Carolina State University, Raleigh, NC 27695 USA

**Keywords:** Agricultural genetics, Functional genomics, Gene expression, Plant breeding, Plant genetics, Transcriptomics, Genomics, Comparative genomics

## Abstract

Pineapple originates from tropical regions in South America and is therefore significantly impacted by cold stress. Periodic cold events in the equatorial regions where pineapple is grown may induce early flowering, also known as precocious flowering, resulting in monetary losses due to small fruit size and the need to make multiple passes for harvesting a single field. Currently, pineapple is one of the most important tropical fruits in the world in terms of consumption, and production losses caused by weather can have major impacts on worldwide exportation potential and economics. To further our understanding of and identify mechanisms for low-temperature tolerance in pineapple, and to identify the relationship between low-temperature stress and flowering time, we report here a transcriptomic analysis of two pineapple genotypes in response to low-temperature stress. Using meristem tissue collected from precocious flowering-susceptible MD2 and precocious flowering-tolerant Dole-17, we performed pairwise comparisons and weighted gene co-expression network analysis (WGCNA) to identify cold stress, genotype, and floral organ development-specific modules. Dole-17 had a greater increase in expression of genes that confer cold tolerance. The results suggested that low temperature stress in Dole-17 plants induces transcriptional changes to adapt and maintain homeostasis. Comparative transcriptomic analysis revealed differences in cuticular wax biosynthesis, carbohydrate accumulation, and vernalization-related gene expression between genotypes. Cold stress induced changes in ethylene and abscisic acid-mediated pathways differentially between genotypes, suggesting that MD2 may be more susceptible to hormone-mediated early flowering. The differentially expressed genes and module hub genes identified in this study are potential candidates for engineering cold tolerance in pineapple to develop new varieties capable of maintaining normal reproduction cycles under cold stress. In addition, a total of 461 core genes involved in the development of reproductive tissues in pineapple were also identified in this study. This research provides an important genomic resource for understanding molecular networks underlying cold stress response and how cold stress affects flowering time in pineapple.

## Introduction

Pineapple is the second most important tropical fruit in terms of international trade and is consumed in both fresh and processed forms (FAO, 2022). Pineapple is grown in warm tropics and subtropics, therefore, periodic cold events (≤ 18 °C) in its growing regions can cause stress, leading to production issues such as precocious flowering^1,2^. Precocious flowering is characterized by stress-induced, asynchronous flowering of same-age plants across a growing field^2–5^.

Several factors contribute to a pineapple plant’s susceptibility to early or precocious natural flowering induction, including plant age, size, carbon to nitrogen (C/N) ratio, responsiveness to ethylene, and stress tolerance^1–3,6,7^. Under stress conditions, flowering can be induced at improper times, resulting in missed harvest and production losses. MD2, the most commonly grown cultivar for commercial export^8–11^, is highly susceptible to precocious flowering, therefore, cold stress-mediated natural induction creates a major bottleneck for pineapple production.

Cold stress is among factors proposed to induce early flowering in pineapple, therefore, some previous work on pineapple includes studies that have attempted to identify genetic mechanisms for cold tolerance^12,13^. Multiple studies have reported the successful alteration of flowering time using chemical and/or cultural practices and through genetic engineering^5,6,14,15^. However, most studies on pineapple floral induction were carried out using hormone treatment. Despite the variety of available research for pineapple, there is a lack of available genomic data for pineapple induced to flower by exposure to periodic cold fronts under natural field conditions. Although current data is helpful, we do not know if results found by studies that rely on ethephon-induced artificial flowering represent the same response that the plant has when precocious flowering is induced by environmental stress, particularly cold. The genetic mechanisms underlying the relationship between cold tolerance and flowering time in pineapple have not yet been elucidated.

Given the lack of data available for cold stress-induced precocious flowering in pineapple, we attempted to fill this gap in the current research. In this study, comparative transcriptome analysis of two types of field-grown pineapple, one susceptible (MD2) and one tolerant (Dole-17) to precocious flowering, subjected to natural cold stress was performed to identify candidate genes related to cold tolerance and potentially precocious flowering. Using samples collected from Dole-17 and MD2 genotypes after 3 natural cold events, as well as before and after floral induction, we identified differentially expressed genes (DEGs) and co-expressed gene modules involved in cold stress response and cold tolerance and were able to identify genes within the DEGs and modules that regulate flowering time in pineapple.

This work contributes to the overall understanding of the genetic mechanisms regulating cold stress response and the vegetative-to-floral transition in pineapple after exposure to cold temperatures. Future work could involve using transgenic or targeted breeding approaches to develop new cold stress-tolerant pineapple varieties that are potentially less prone to precocious flowering.

## Results

### Phenotypic response of two pineapple varieties to cold stress

**Figure 1:**
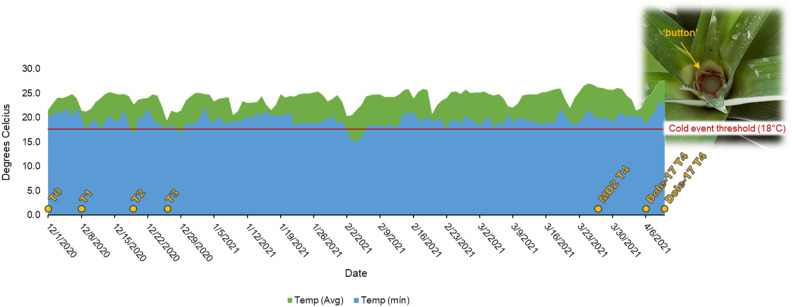
Visual representation of experimental design and flowering time phenotype results in this study. A cold event was considered as temperature of ≤ 18 °C for at least 12 h. The dates (x-axis) begin on the first day of sample collection (December 1, 2020) and end on the last day of sample collection (April 10, 2021). Three biological replicates were collected for all samples (Dole-17 and MD2 genotypes; time points T0–T4). Flowering (T4) samples were collected upon the first visual sign of floral development (button stage).

Notable differences in flowering time were observed for the two genotypes used in this study. The first signs of flowering were observed up to two weeks later in Dole-17 (precocious flowering tolerant) than in MD2 (precocious flowering susceptible) (Supplementary Figure S1). As expected, after three cold events (see materials and methods), the button stage (T4) was visible much earlier in MD2 on March 27, 2021, while it was not visible in Dole-17 until April 6–10, 2021 (Fig. 1).

### Comparative transcriptional response of two pineapple genotypes to cold stress

For this study, we performed multiple comparisons, including between time points (e.g., Dole-17_T0_ vs Dole-17_T1_), as well as between genotypes (e.g., Dole-17_T0_ vs MD2_T0_). The largest differences in expression were observed between genotypes (e.g., Dole-17_T1_ vs MD2_T1_), as opposed to between time points (e.g., Dole-17_T0_ vs Dole-17_T1_) (Table 1). DEGs identified from comparisons between genotypes and between time points T0 through T3 were mined for stress-related genes and DEGs specific to the T4 time point were core genes involved in floral organ development (Supplementary Figure S2).

**Table 1:** Differentially expressed gene (DEG) matrix depicting the number of genes with significantly different expression levels between samples in pairwise comparisons. In this study, T0 represents field-grown pineapple plants before any annual cold fronts; T1, T2, and T3 represent plants after 1, 2, and 3 cold events, respectively; and T4 represents plants in the earliest visible stages of flowering.

	Dole17 T0	Dole17 T1	Dole17 T2	Dole17 T3	Dole17 T4	MD2 T0	MD2 T1	MD2 T2	MD2 T3	MD2 T4
Dole17 T0	0	84 (79/5)	175 (170/5)	65 (64/1)	1,142 (500/642)	1,293 (207/1086)	1,542 (400/1,142)	1,545 (447/1,098)	1,612 (544/1,068)	2,402 (698/1,704)
Dole17 T1	84	0	42 (16/26)	70 (18/52)	1,166 (465/701)	1,398 (180/1,218)	1,353 (185/1,168)	1,423 (232/1,191)	1,396 (287/1,109)	2,383 (622/1,761)
Dole17 T2	175	42	0	24 (2/22)	1,229 (351/878)	1,426 (172/1,254)	1,435 (214/1,221)	1,319 (182/1,137)	1,340 (232/1,108)	2,367 (487/1,880)
Dole17 T3	65	70	24	0	1,193 (354/839)	1,348 (148/1,200)	1,460 (226/1,234)	1,370 (214/1,156)	1,326 (237/1,089)	2,351 (481/1,870)
Dole17 T4	1,142	1,166	1,229	1,193	0	2,512 (923/1,589)	2,679 (1,035/1,644)	2,603 (1,074/1,529)	2,680 (1,185/1,495)	1,492 (311/1,181)
MD2 T0	1,293	1,398	1,426	1,348	2,512	0	72 (67/5)	86 (82/4)	76 (76/0)	1,154 (445/709)
MD2 T1	1,542	1,353	1,435	1,460	2,679	72	0	10 (1/9)	17 (13/4)	1,418 (559/859)
MD2 T2	1,545	1,423	1,319	1,370	2,603	86	10	0	10 (8/2)	1,301 (433/868)
MD2 T3	1,612	1,396	1,340	1,326	2,680	76	17	10	0	1,228 (335/893)
MD2 T4	2,402	2,383	2,367	2,351	1,492	1,154	1,418	1,301	1,228	0

Former numbers in parentheses represent number of up-regulated genes and latter numbers in parentheses represent number of down-regulated genes in the sample comparison.

GO enrichment analysis indicated genes involved in regulatory functions were enriched after cold treatment for both genotypes (Supplementary Table S1) and some of these regulatory genes demonstrated genotype-specific expression therefore, the regulatory gene families represented in each genotype were further examined. Dole-17 and MD2 had contrasting profiles of differentially expressed regulatory gene families involved in abiotic stress response and flowering time, including transcription factors (TFs) and transcription regulators (TRs) in *A20, AP2/ERF, bHLH, bZIP, MYB, C2C2-GATA, C2H2, NAM, WRKY*, and *TIFY* gene families^7,12^. Dole-17 had more *A20, C2H2, MYB, bZIP*, and *WRKY* DEGs, while MD2 had more *AP2/ERF* and *bHLH* DEGs; both genotypes also shared DEGs representing TF and TR gene families *A20, AP2/ERF, bHLH, C2C2-GATA, MYB, NAM, TIFY*, and *WRKY*.

Further examination of specific regulatory DEGs provided insight into the differences in abiotic stress tolerance and regulation of flowering time between Dole-17 and MD2 genotypes. For example, *RAV1*, an AP2/ERF domain-containing TF that negatively regulates flowering^16,17^ was up-regulated in Dole-17_T0_ and was the only pineapple flowering ortholog that was differentially expressed between time points (Supplementary Tables S2 and S3). A *bHLH* TF that positively regulates flowering (*bHLH63*)^16^ was up-regulated in the MD2 T0 time point, while another *bHLH* TF (*bHLH35*) previously found to improve cold tolerance in *Arabidopsis*^18^, was up-regulated in the Dole-17_T0_ time point.

Next, we examined comparisons between genotypes at each time point (T0-T3). MD2 had 4.5 to 6.3-fold more up-regulated genes than Dole-17 for T0 to T3 time points (Table 1). A total of 103 DEGs were consistently up-regulated in Dole-17 (down in MD2) across all T0 through T3 time points, while 939 were consistently down-regulated in Dole-17 (up in MD2) across all T0 through T3 time points (Supplementary Figure S3). Functional enrichment analysis of this subset of DEGs confirmed that “GO:0006950 response to stress” and “GO:0006952 defense response” are among the most significantly enriched biological processes for all time points from T0 to T3.

Several cysteine-rich receptor-like kinases (*CRKs*) and cytochrome P450s (*CYPs*) were consistently up-regulated in Dole-17 compared to MD2 across all T0 to T3 time points. *CRKs* and *CYPs* play a major role in adaptation to biotic and abiotic stress in plants^19,20^, and *CYPs* specifically function in flavonoid biosynthesis, including anthocyanin, which can help plants tolerate stress^21^. Other genes that were up-regulated in Dole-17 across all T0 to T3 time points included those encoding temperature and dehydration-induced proteins, as well as proteins in the photoperiodic flowering pathway. Low-temperature induced proteins and heat shock proteins have previously been implicated in cold stress response and adaptation in plants^22,23^ (Supplementary Table S3).

Consistently down-regulated DEGs in Dole-17 included several regulatory gene families involved in vernalization and stress hormone-mediated signaling, including *AP2/ERF*^17,24,25^, *bHLH*^26^, *C3H-WRC/GRF*^27^, *FRS/FRF*^11^, *GRAS*^28^, *MADS-MIKC*^29,30^, *JmjC*^30^, *SBP*^31–33^, and *SET*^34^. Ethylene has been identified as the major hormone promoting flowering in pineapple^1,15^, therefore, the consistent down-regulation of ethylene-responsive TFs (*AP2/ERF*) in Dole-17 likely played a role in the observed differences in flowering time between Dole-17 and MD2. In addition, the cross-talk between cold stress response and ethylene signaling has been previously reported for pineapple ^12,35^, therefore, there may also be a link between cold tolerance and ethylene production and/or sensitivity in pineapple.

Other genes in the consistently down-regulated subset included genes in the vernalization pathway, such as a *FRIGIDA-like protein* (*FRI3*)^32,36,37^, and flowering genes identified by orthology. It is worth noting that all pineapple flowering orthologs that were consistently DEGs between genotypes (7) were down-regulated in Dole-17, suggesting that the cold event in MD2 induced the over expression of flowering genes. Some of these orthologs included flowering time control protein FY, a positive regulator of flowering transition in the autonomous pathway^36^, a SET-domain-encoding gene (*CLF*) that interacts with *FRI* to control flowering time via the vernalization pathway in many plants^36,38^, a mitochondrial carrier *MTM1-like* protein that participates in flowering time control^39^, a phosphatidylinositol 4-kinase gamma 4-like protein that plays a role in florigen transport and delays flowering in rice^40^, and a receptor-like kinase (RLK) *THESEUS 1* (*THE1*) protein, a member of a family of RLKs that function in the regulation of many biological processes including stress response and flowering time^41,42^ (Supplementary Table S3).

In addition, 9 and 37 DEGs were up- and down-regulated, respectively, between Dole-17 and MD2 genotypes across all cold event time points (T1, T2, T3), but were not differentially expressed between genotypes at T0. The function of these genes was further investigated to determine their potential roles in cold response. DEGs that were up-regulated in Dole-17 after all 3 cold events included genes that function in abiotic stress signaling, cold tolerance, and floral organ development. For example, genes encoding a chalcone synthase enzyme (*CHS*), a Kelch domain-containing F-box protein, a *cytochrome P450 78A9-like* protein (*CYP78A9*), and a rice *GLOSSY-1* (*GL1*)/*Arabidopsis ECERIFERUM 3* (*CER3*) homolog (Supplementary Table S3). *CHS* can be induced by abiotic stress, including low temperature, in several species, leading to the production of anthocyanins^43^ and Kelch domain-containing F-box proteins physically interact with *CHS* in the flavonoid biosynthesis pathway^44^. *CYP78A9* is down-regulated by chalcone isomerase^45^, a key enzyme involved in anthocyanin biosynthesis^46^. The synthesis of flavonoids such as anthocyanins can function as protection against abiotic stresses and the accumulation of reactive oxygen species (ROS), thereby reducing ROS-mediated damage to cells^47^. A mechanism that plants use to minimize cold stress is to induce cuticular wax production that helps to prevent frost damage and reduces water loss^48^. *GL1* in rice (synonymous with *CER3* in *Arabidopsis*) is associated with increased wax production^48,49^, therefore, the up-regulation of this gene in Dole-17 may indicate that cold tolerant pineapple varieties might use the same mechanism to reduce cold damage to plant tissues. Indeed, previous studies also found that cell wall biosynthesis was associated with increased tolerance to cold stress in pineapple^12^.

DEGs that were down-regulated in Dole-17 after all 3 cold events included a *cytochrome P450 78A11* (*CYP78A11*), a sucrose transporter (*SUT1*), and a *FAR1/FRS* TF (Supplementary Table S3). Functional annotation of these genes using GO terms revealed they are involved in floral transition and nutrient accumulation. *CYP78A11* is a vegetative phase timekeeper in rice and is involved in the transition to the reproductive phase^50^, *SUT1* has been shown to be expressed in pollen and is necessary for normal pollen function in rice^51^, and finally, *FAR1/FRS* family TFs have been previously implicated in pineapple flowering due to their role in red and far-red light response^11^, which is a photoperiodic trigger to flower for many plant species. The function of these genes combined with them being up-regulated in MD2 suggested that they may play some role in the earlier flowering time phenotype observed in MD2.

**Figure 2:**
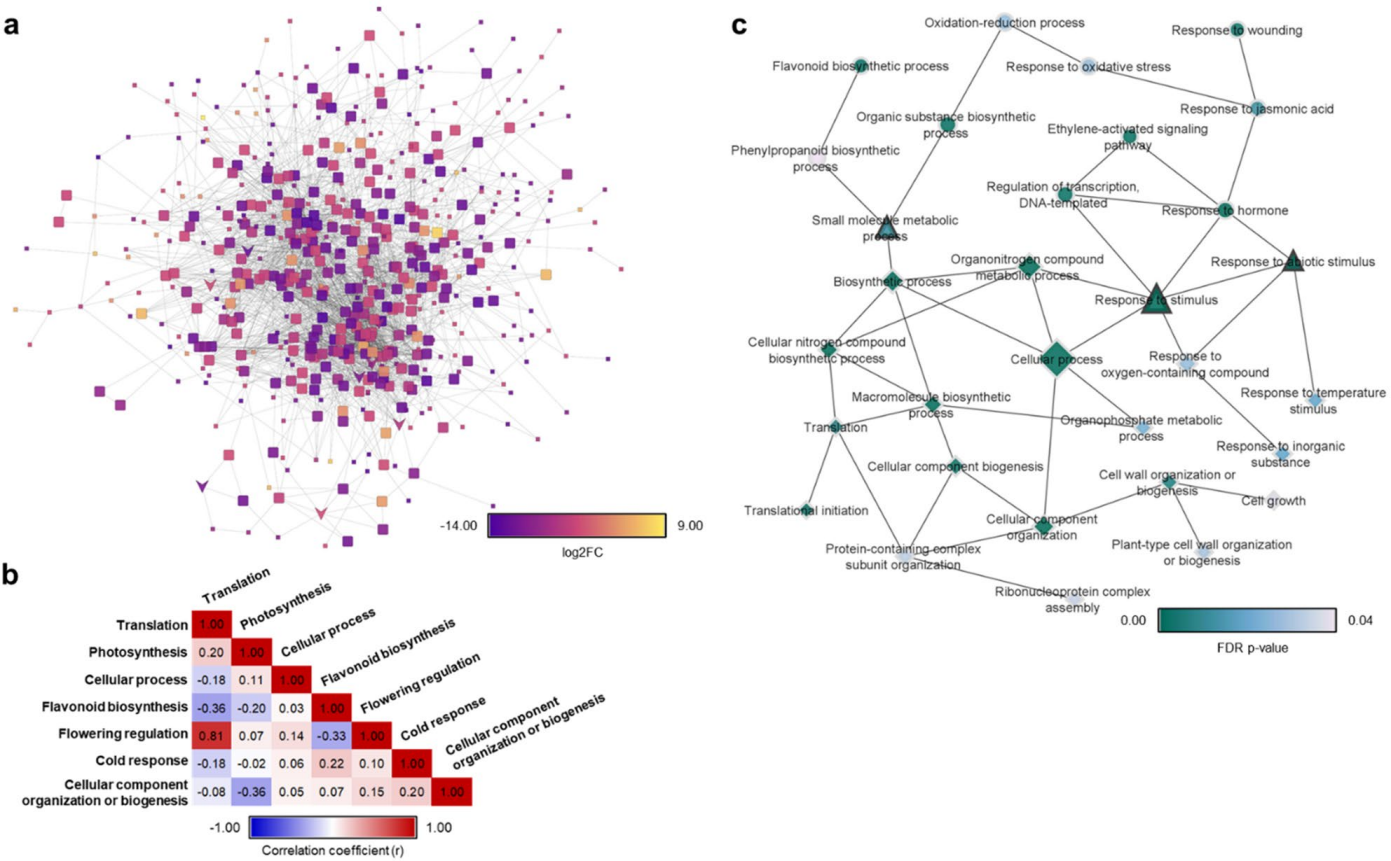
Interactome analysis results using DEGs between Dole-17 and MD2 at T1, T2, and T3 time points. (**a**) Gene interaction network of DEGs. Node color corresponds to Log2FC expression level between Dole-17 vs MD2, and node shape corresponds to MCODE cluster status (V = seed, square = clustered, small square = unclustered); (**b**) Pearson’s correlation results using gene expression values from GO term enriched MCODE clusters; (**c**) Enriched processes in the set of DEGs used for interactome analysis. Node shape corresponds to expression in Dole-17 compared to MD2 (up-regulated = circle, down-regulated = diamond, shared = triangle), node size corresponds to the number of genes, and node color corresponds to significance level.

Interactome analysis of the subset of DEGs between genotypes at T1, T2, and T3 time points (see materials and methods) confirmed that significant differences in expression of stress-mitigating genes occurred between genotypes after experiencing cold stress (Fig. 2a). A Pearson’s correlation network built using gene expression patterns indicated a positive correlation between cold stress response and biosynthesis of flavonoids, flowering regulation, and cell component biogenesis, while expression patterns indicated a negative correlation between cold stress response and photosynthesis and protein translation (Fig. 2b). GO enrichment analysis of the subset of DEGs further validated that in Dole-17, stress-mitigating genes were up-regulated while genes involved in growth and development were down-regulated after cold stress (Fig. 2c).

Overall, the DEGs identified between genotypes and between time points were clearly related to cold-response and these results provided some initial insight into the mechanism underlying cold stress response in pineapple. However, we wanted to further investigate the relationships between genes that function in cold stress response and their potential link to flowering time regulation.

### Identification of co-expressed gene modules

Identification and functional annotation of co-expressed gene modules was used to determine sets of genes with similar patterns of expression that are likely to function in cold stress response. Modules associated with cold stress response were evaluated for their overlap with DEGs and to determine their biological role more specifically. Regulatory genes (e.g., TFs and TRs, etc.) and hub genes were also identified in the stress response-related modules in order to determine key regulatory genes involved in stress response. Finally, because pineapple has been known to prematurely flower and set fruit after natural cold fronts in their growing region^4,6,12,13,52^, flowering orthologs were also identified in the stress-related co-expression modules to examine the relationship between cold stress response and flowering in pineapple.

Out of all modules identified using expression data from T0-T3 samples, 10 were selected for further analysis because of their eigengene expression patterns (Table 2). These modules were correlated with genotype, time point, and/or condition (Supplementary Figure S4). Out of these, 8 modules were associated with stress response and 2 modules were associated with consistent differences between genotypes. In a separate analysis using expression data from T0-T4 samples, 3 modules were associated specifically with the T4 timepoint (Table 2).

**Table 2:** Statistics for gene co-expression modules identified in this study.

Analysis	Module	Association	Total genes (DEGs*)	Hub genes*^, ^**	Regulatory genes*	Flowering orthologs*	GO:0009409 response to cold*
WGCNA Analysis1	Turquoise	Genotype	8,591 (1,336)	1,739 (1,100)	910 (139)	70 (8)	100 (19)
	Blue	Genotype	5,083 (250)	811 (113)	528 (22)	37 (0)	63 (7)
	Grey60	Cold response	3,255 (184)	4 (1)	422 (55)	20 (1)	43 (4)
	Lightyellow	Cold response	283 (60)	36 (15)	38 (13)	4 (0)	4 (2)
	Yellow	Cold response	1,475 (11)	8 (0)	247 (1)	25 (0)	14 (0)
	Black	Cold response	1,124 (10)	35 (0)	135 (1)	21 (0)	16 (0)
	Red	Cold response	977 (21)	70 (3)	111 (1)	6 (0)	7 (0)
	Purple	Cold response	409 (9)	19 (0)	66 (1)	3 (0)	6 (0)
	Greenyellow	Cold response	364 (0)	32 (0)	45 (0)	1 (0)	2 (0)
	Darkturquoise	Cold response	166 (0)	3 (0)	19 (0)	2 (0)	3 (0)
WGCNA Analysis2	Blue	Time point T4	5,541 (1,185)	188 (117)	642 (162)	41 (11)	82 (25)
	Royalblue	Time point T4	560 (120)	10 (2)	85 (27)	4 (2)	3 (1)
	Sienna3	Time point T4	1,609 (178)	16 (5)	229 (44)	28 (3)	17 (3)

*Only refers to DEGs relevant to the WGCNA Analysis (i.e., WGCNA1 included T0-T3 and WGCNA2 included T0-T4).

**Hub genes determined by gene significance value ≥ │0.5│and module membership value ≥ 0.9, except when modules are smaller, values were slightly reduced.

Parentheses () indicates number of DEGs in the respective category.

**Figure 3:**
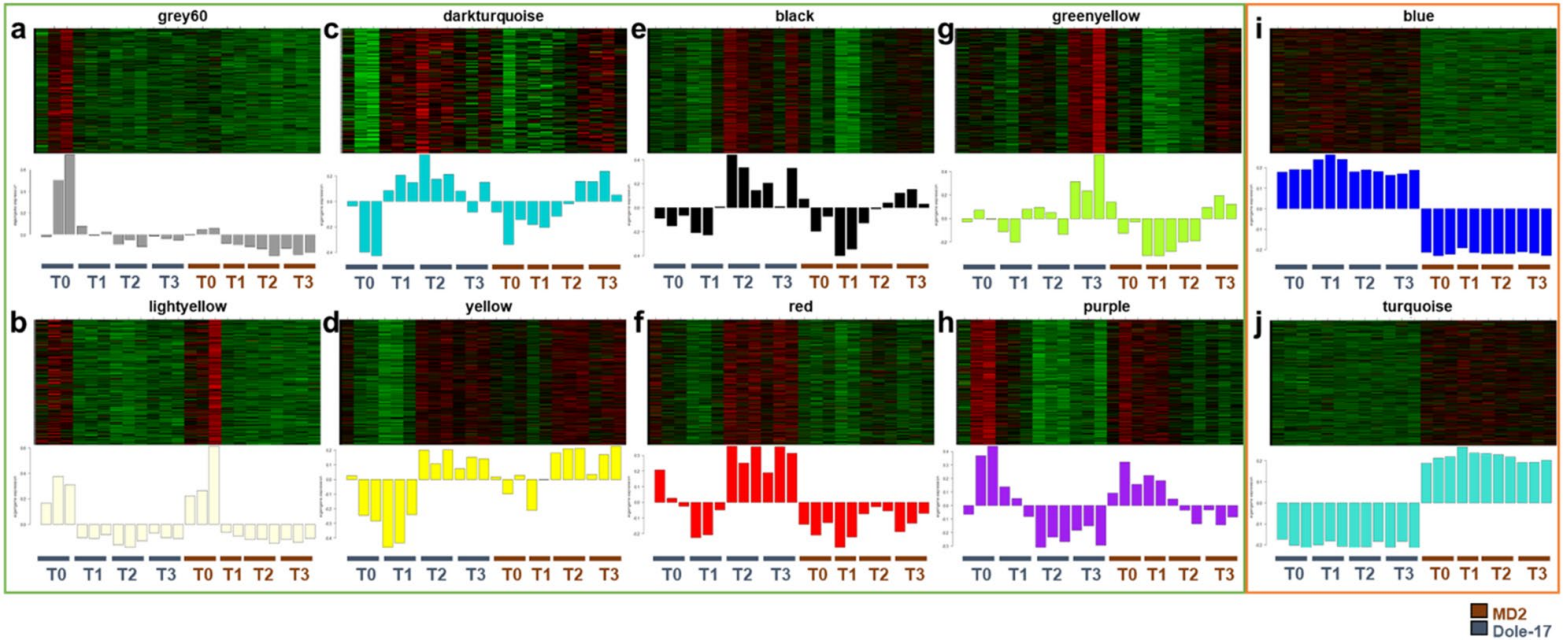
Heatmap and bar plots for (**a**) grey60, (**b**) lightyellow, (**c**) darkturquoise, (**d**) yellow, (**e**) black, and (**f**) red, (**g**) greenyellow, (**h**) purple, (**i**) blue, and (**j**) turquoise co-expression modules. Top panels include expression heatmaps for all genes in the module and bottom panels include eigengene expression level bar plots. Eigengene expression (y-axis on bar plots) refers to the representative gene expression profile of a module. Modules within the green rectangle were involved in cold response and modules within the orange rectangle were involved in genotype-specific gene expression.

The grey60 module (3,255 genes) was a large module composed of genes with high levels of relative expression in the Dole-17_T0_ time point (Table 2; Fig. 3a). One hundred and eighty-four total DEGs were found within this module, which were generally either up-regulated in Dole-17_T0_ compared to Dole-17_T1-T3,_ and MD2_T0_, or were up-regulated in Dole-17_T3_ compared to MD2_T3_. Additionally, out of all DE TFs identified in the grey60 module, *AP2/ERF* was the largest family (12 genes including *RAV-type AP2/ERF* TFs). This family of TFs play a major role in ethylene signaling in pineapple^17^ (Supplementary Table S4). Other DE TFs, including *bHLH, C2H2, MYB, HSF*, and *WRKY* were also identified in the grey60 module.

The lightyellow module (283 genes) also contained genes primarily up-regulated in the T0 time point (Table 2; Fig. 3b), however, unlike the grey60 module, this up-regulation was in the T0 time point for both Dole-17 and MD2 genotypes. The lightyellow module also contained similar TF families as the grey60 module (Supplementary Table S4), however, this module also contained two DEGs encoding A20/AN1 domain-containing TRs (Supplementary Table S3).

GO enrichment of the grey60 and lightyellow modules confirmed that the genes in these modules function in regulating abiotic stress responses and early hormone signaling. For example, biological process GO terms “GO:0009611 response to wounding” and “GO:0071395 cellular response to jasmonic acid stimulus” were over-represented in the grey60 module, while GO terms “GO:0042218 1-aminocyclopropane-1-carboxylate biosynthetic process” and “GO:0070413 trehalose metabolism in response to stress” were over-represented in the lightyellow module.

The darkturquoise module was the smallest module identified in this study (Table 2), containing only 166 genes. An eigengene expression plot suggested that genes in this module were affected by and responded to cold treatment (Fig. 3c). These genes had low levels of expression at T0 for both Dole-17 and MD2 genotypes, but after the first cold event (T1), gene expression increased in Dole-17 and not MD2. After the second cold event (T2), gene expression increased in both genotypes and were at their highest expression level in Dole-17_T2_. While there were no DEGs identified in this module, it did contain 3 hub genes, 19 regulatory genes, and 2 flowering orthologs (Table 2). Enrichment analysis of the darkturquoise module indicated that these genes function in epigenetic regulation of gene and protein expression; enriched biological process GO terms included “GO:0006413 translational initiation”, “GO:0006325 chromatin organization”, “GO:0035196 miRNA processing”, and “GO:0035195 miRNA-mediated gene silencing”. Cold response annotated genes in the darkturquoise module included genes involved in mRNA splicing^53^, miRNA-mediated gene silencing and pollen development^54,55^, ABA signaling and post-translational modification of circadian clock proteins^56,57^. Epigenetic regulation of mRNA and protein levels in response to abiotic stress plays a role in regulating flowering time^58^. Hub genes in this module included genes involved in altering plant morphology and have previously been reported to respond to various abiotic stresses^59,60^. These results suggest that genes in this module likely play a role in responding to cold stress and mediating accumulation of flowering-related proteins.

The yellow module was the largest module (1,475 genes) (Table 2) identified as likely being associated with cold stress response and represented genes with low expression in Dole-17_T0_ and very low expression in Dole-17_T1_ relative to all other samples (Fig. 3d). In addition, large differences in gene expression were observed between Dole-17_T1_ and Dole-17_T2_, indicating that this module likely plays a role in responding to cold stress. Enriched biological process GO terms for the yellow module included “GO:2000035 regulation of stem cell division”, “GO:0048510 regulation of timing of transition from vegetative to reproductive phase”, and “GO:0010154 fruit development”, suggesting that this module may be important for reproduction in pineapple. GO annotation of yellow module hub genes also suggested that this module functions in cell division and response to auxin, ethylene, and SA which are major inducers of flowering in pineapple^4,15,17,61^, and similar responses have been observed in other studies on cold stress in pineapple^12,13^ and in comparative studies on ethylene tolerance in pineapple^7^.

The black module contained 1,124 genes (Table 2) that displayed significant differences in expression between Dole-17 and MD2 at T1, T2, and T3 time points (Fig. 3e). Black module genes exhibited down-regulation in MD2_T1_ and up-regulation in Dole-17_T2_ relative to all other samples. The DE analysis results also reflected the eigengene expression plot, as the DEGs (10 genes) in the black module were up-regulated in Dole-17 compared to MD2 at the T1, T2, and T3 time points. Functional annotation of DEGs in the black module indicated that this module functions in cold-induced cell wall thickening and morphological development that is likely associated with vegetative to floral phase change. Several transcriptional regulatory gene families were represented in the black module (Supplementary Table S1), however only 1 of those genes was DE, a *WUSCHEL-related homeobox* (*WOX*) family TF homologous to BEL1-like homeodomain 4 (*BLH4*). The *BLH4* gene was up-regulated in Dole-17_T2_ and MD2_T0_ compared to MD2_T2_. The role of *BLH4* in stress response and regulating morphological changes, including inflorescence meristem development and cell wall biosynthesis has been widely documented^62^ (Supplementary Table S3). Other DEGs within the black module included the GL1-1 homolog mentioned previously that functions in cuticular wax biosynthesis^49^ and other genes have been previously shown to function in plant growth, cell wall development, and conferring cold tolerance^63–66^. GO enrichment analysis further supported that the genes in this module function in response to stress, epigenetic regulation of floral transition, and organ development. Biological process GO terms enriched in the black module included “GO:0031058 positive regulation of histone modification”, “GO:0033554 cellular response to stress”, and “GO:0051301 cell division”. One of the 35 hub genes in the black module (*ACMD2v2_12.29369*) was both a flowering ortholog and a cold response gene, and encoded a Polycomb group (PcG) protein (*FIE2*), which plays a role in epigenetic-regulated floral transition in the vernalization pathway^36,67^ (Supplementary Table S3).

The red module contained 977 genes (Table 2) with relatively high expression levels in Dole-17_T2-T3_ time points compared to all other samples (Fig. 3f). The expression levels across T0 to T3 time points were also genotype-dependent, with Dole-17 and MD2 having distinct expression patterns. Given the expression patterns observed for the red module, we hypothesized that this module may play an important role in cold stress responses in Dole-17. Indeed, GO term enrichment of this module indicated that these genes likely function in RNA and protein processing and transport in response to abiotic stress. Enriched biological process GO terms in this module included “GO:0016926 protein desumoylation”, “GO:0009451 RNA modification” and “GO:0031503 protein-containing complex localization”. Three genes that were DEGs were also hub genes in the red module, two of which could be novel genes involved in cold tolerance. One of the hub DEGs was orthologous to a Myb/SANT-like domain-containing protein in rice, which may be involved in repression of protein translation^68^, another hub DEG was a putative CCCH-type zinc finger family protein, which have been previously reported to function in a wide range of plant developmental processes, including flowering^30,69^ and ABA response^70^ (Supplementary Table S3). And the last hub DEG was not able to be fully annotated based on sequence homology, however, it did contain a CCHC-type zinc finger domain. Based on previous studies of CCHC(Zn) genes, this gene in pineapple may be involved in abiotic stress response^71^. These results indicate that it could be worthwhile to investigate the function of these 3 genes in future research.

The greenyellow module was made up of 364 genes (Table 2) with relatively high expression in the T3 time point compared to other time points (T0-T2) for both genotypes (Fig. 3g), but Dole-17 had higher gene expression than MD2 for each time point (T0-T3). Enriched GO terms in this module included “GO:0000278 mitotic cell cycle”, “GO:0007010 cytoskeleton organization” and “GO:0051321 meiotic cell cycle”, indicating that this module is likely involved in cell division and organ development. Hub genes in the greenyellow module included GO term annotations associated with mitosis, meiosis, and epigenetic regulation of gene expression, providing support that this module is important in plant organ development. Two cold response genes in this module were *HK3* and *TUB6*, and the only flowering ortholog was *CLF* (Supplementary Table S3). *HK3* is a cytokinin receptor involved in inducing cell division and regulation of meristem development, and has been experimentally shown to negatively regulate ABA signaling and cold stress responses^72^. *TUB6* encodes a β-tubulin gene involved in cytoskeleton organization and mitosis, and has been reported to be cold-responsive in *Arabidopsis*^73^. As mentioned previously, *CLF* plays a key role in transcriptional silencing of *FLC* during vernalization, resulting in transition from the vegetative to reproductive phase^36,38,74^. Given the presence of the genes discussed here in the greenyellow module, combined with the gene expression patterns seen in this module, we can speculate that this module may be involved in floral meristem organogenesis in pineapple.

The purple module contained 409 genes (Table 2) and eigengene expression patterns exhibited relatively low expression levels in Dole-17_T2-T3_ time points (Fig. 3h). In addition, Dole-17 had greater changes in gene expression between time points (e.g., from T0 to T1, T1 to T2, etc.) than MD2. No biological process GO terms were enriched in the purple module gene set, however, enriched cellular component GO terms included “GO:0005784 Sec61 translocon complex”, therefore, this module may be involved in cold-induced protein secretion and transport. Nine DEGs identified in this module were primarily down-regulated in Dole-17_T2_ compared to Dole-17_T0_ and they included a *CRF2-like* TF gene, which plays an important role in maintenance of root system architecture and is induced by cold temperatures in *Arabidopsis*^75^. Six cold response genes were in the purple module (Supplementary Table S3), one of which was an ABA receptor (*PYL8*). Like *CRF2*, *PYL8* promotes lateral root growth under abiotic stress conditions^76^. Three out of the 19 hub genes in the purple module were regulatory genes, including two C2H2-type zinc finger TFs (*MBS1* and *ATL79*) and a squamosa promoter-binding-like protein (*SPL3*), and one was a cold response gene, a PLAT domain-containing protein (*PLAT3*). *MBS1* plays a role in ROS signaling and regulating plant growth during stress^77^ and *ATL79* functions in protein processing and degradation during abiotic stress^78^. *SPL3* is a flowering-promoting TF that can be repressed by *FAR1/FRS* family TFs and by DELLA proteins^79^. PLAT domain proteins are positive regulators of abiotic stress tolerance and promote growth under normal, non-stressful conditions^80^. These results suggest that the purple module may play an important role in regulating growth and development during periods of abiotic stress.

### Genotype-specific modules

The two largest modules identified in the WGCNA analysis of T0-T3 time points, the blue (8,591 genes) and turquoise (5,083 genes) modules (Table 2), were associated with genotype-specific gene expression. Eigengene expression in the blue module displayed up-regulation of gene expression in Dole-17 compared to MD2 for all time points, and vice-versa in the turquoise module (Fig. 3i,j). Expression patterns of genes that were consistently identified as DEGs between genotypes across T0-T3 time points strongly overlapped with eigengene expression patterns in the blue and turquoise modules.

Functional enrichment analysis of the genotype-specific modules demonstrated that Dole-17 and MD2 had major differences in expression of stress-related genes. Enriched biological process GO terms in the blue module included “GO:0051641 cellular localization” and “GO:0044085 cellular component biogenesis”, while enriched biological process GO terms in the turquoise module included “GO:2000306 positive regulation of photomorphogenesis” and “GO:0006950 response to stress”.

The blue module had 63, while the turquoise module had 100 genes functionally annotated with the GO term “GO:0009409 response to cold” (Table 2). In addition, 3 of the cold response DEGs in the blue module and 17 of the cold response DEGs in the turquoise module were hub genes, which demonstrates that cold responsiveness was a key driver of both of these modules. Both blue and turquoise modules also had flowering orthologs as hub genes, demonstrating that, in addition to cold responsiveness, flowering regulation was a key driver for these modules. The differences in genotype-specific expression observed here provide some reasoning as to why Dole-17 displays a later flowering time than MD2 after cold stress.

### Gene expression validation with RT-qPCR

Eleven genes (*ACMD2v2_01.04652*, *ACMD2v2_02.24982*, *ACMD2v2_02.26328*, *ACMD2v2_03.17852*, *ACMD2v2_03.18264*, *ACMD2v2_06.24315*, *ACMD2v2_10.09091*, *ACMD2v2_10.09239*, *ACMD2v2_14.15670*, *ACMD2v2_18.14935*, *ACMD2v2_20.14300*) were chosen for RT-qPCR to validate the RNA-seq results (Supplementary Tables S3 and S5). These were DEGs potentially involved in the transition to flowering based on functional annotation.

According to the RNA-seq data, genes *ACMD2v2_03.18264*, *ACMD2v2_14.15670*, *ACMD2v2_18.14935*, and *ACMD2v2_20.14300* were DE between Dole-17 and MD2 genotypes at the T0-T3 time points. Gene *ACMD2v2_01.04652* was DE between T0 and T1, T2, and T3 time points within the Dole-17 genotype. Genes *ACMD2v2_02.26328*, *ACMD2v2_03.17852*, and *ACMD2v2_10.09091* were DE between multiple time points within genotypes and DE between genotypes at the T3 time point. Genes *ACMD2v2_02.24982*, *ACMD2v2_06.24315*, and *ACMD2v2_10.09239* were DE between T4 and T1-T3 time points in both genotypes.

Statistical analysis of the results revealed that time point was a significant source of variation in gene expression for all eleven genes analyzed (Supplementary Tables S6 and S7) and genotype was a significant source of variation in expression for six out of the eleven genes (Supplementary Tables S6 and S8). Fold-change patterns in the RT-qPCR analysis were similar to expression patterns observed in the differential expression analysis.

**Figure 4:**
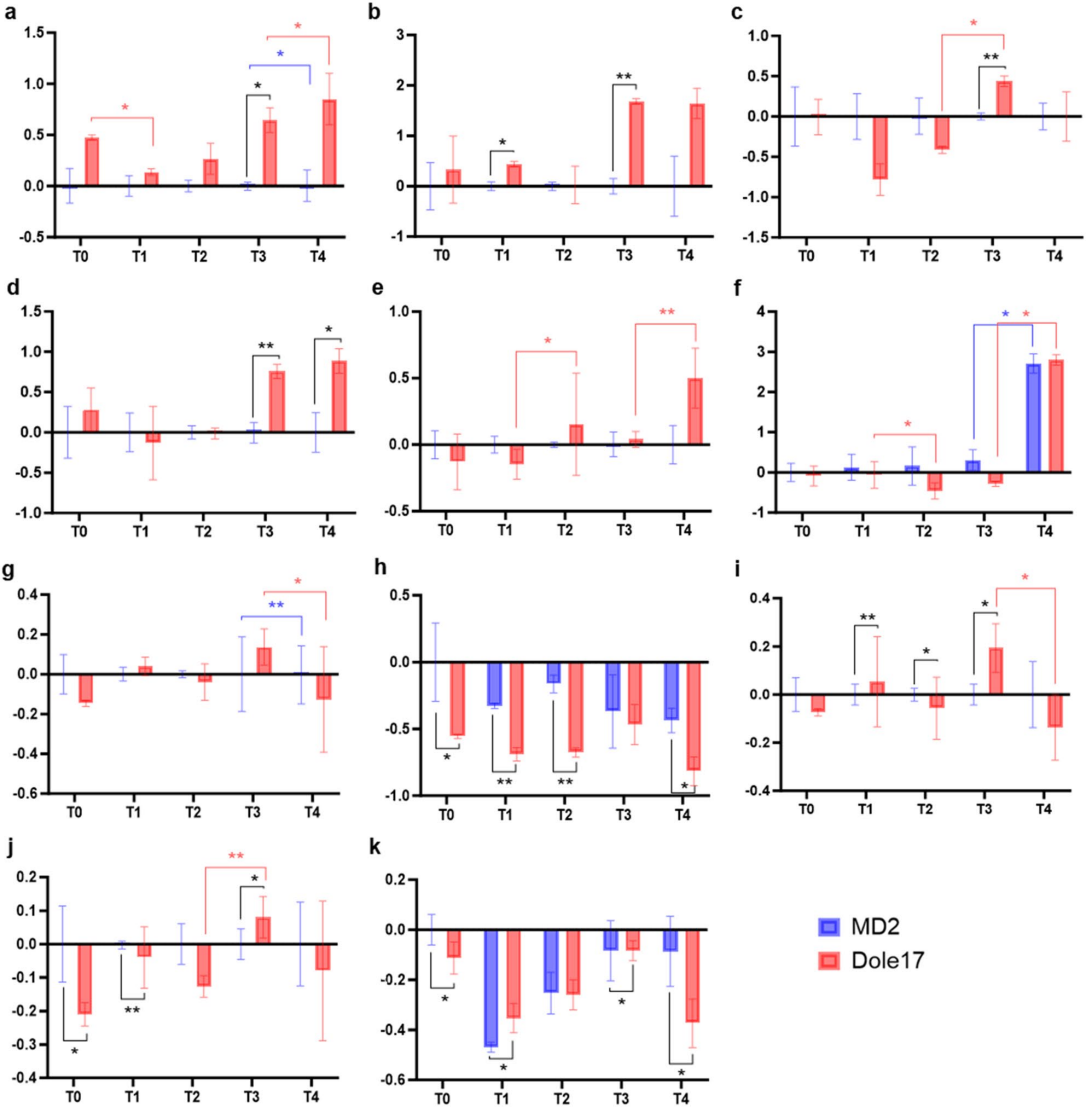
Relative expression results for RT-qPCR genes (**a**) *ACMD2v2_01.04652*, (**b**) *ACMD2v2_02.26328*, (**c**) *ACMD2v2_03.17852*, (**d**) *ACMD2v2_10.09091*, (**e**) *ACMD2v2_02.24982*, (**f**) *ACMD2v2_10.09239*, (**g**) *ACMD2v2_06.24315*, (**h**) *ACMD2v2_03.18264*, (**i**) *ACMD2v2_14.15670*, (**j**) *ACMD2v2_18.14935*, and (**k**) *ACMD2v2_20.14300*. The x-axis represents sample collection time points and the y-axis represents fold change. The bars represent the SD of fold change for three biological replicates. Statistically significant differences between genotypes and time points are indicated with asterisks (“*” = *p*-value < 0.05, “**” = *p*-value < 0.01).

RT-qPCR results confirmed that significant differences in expression existed between genotypes for genes *ACMD2v2_01.04652*, *ACMD2v2_02.26328*, *ACMD2v2_03.17852*, *ACMD2v2_10.09091* (Fig. 4a–d) and between time points for genes *ACMD2v2_01.04652*, *ACMD2v2_02.24982*, *ACMD2v2_03.17852*, *ACMD2v2_06.24315*, *ACMD2v2_10.09239*, *ACMD2v2_14.15670*, and *ACMD2v2_18.14935*. Genes *ACMD2v2_02.24982* and *ACMD2v2_10.09239* had similar expression patterns in the RNA-seq and RT-qPCR analyses (Fig. 4e,f), being strongly up-regulated in the T4 time point, however, *ACMD2v2_02.24982* was up-regulated in T4 for both genotypes in the RNA-seq analysis while the RT-qPCR analysis only revealed up-regulation of expression in Dole-17. Gene *ACMD2v2_06.24315* also shared similar patterns between RNA-seq and RT-qPCR analyses, being down-regulated at the T4 time point in both genotypes (Fig. 4g).

Finally, significant differences in expression of genes *ACMD2v2_03.18264*, *ACMD2v2_14.15670*, *ACMD2v2_18.14935*, and *ACMD2v2_20.14300* existed between genotypes for time points T0-T3 in the RNA-seq results, which were also observed for most of the T0-T3 time points in the RT-qPCR results (Fig. 4h–k). Overall, these results validated the expression patterns observed in the differential expression and co-expression network analyses.

### Pineapple floral organ development

**Figure 5:**
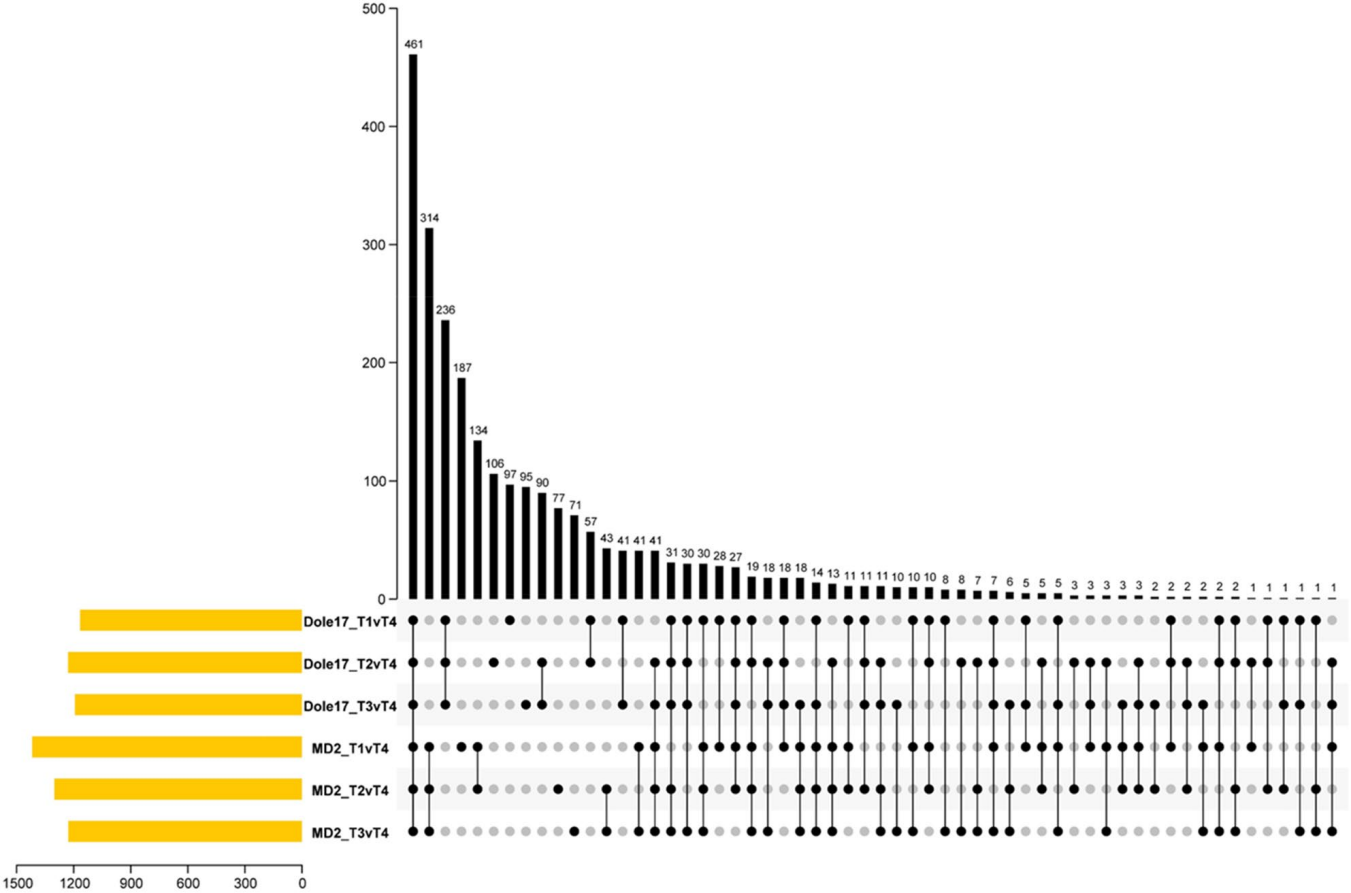
Upset plot depicting differentially expressed gene (DEG) overlap for comparisons between T4 versus cold event (T1, T2, T3) time points in each genotype (Dole-17 and MD2). A total of 461 DEGs were identified as core genes involved in reproductive organ development.

Transcriptome data at T4, representing early flower formation were used to determine which genes are responsible for development of floral organs in pineapple (Table 1). Overlap analysis revealed 461 common DEGs among pairwise comparisons between T4 versus T1, T2, and T3 time points for both genotypes (Fig. 5), which were considered as likely core genes involved in development of reproductive tissues in pineapple. Indeed, GO enrichment analysis indicated that these genes are involved in biological processes related to reproductive structure development, including “GO:0010582 floral meristem determinacy”, “GO:0048834 specification of petal number”, and “GO:0010254 nectary development” (Supplementary Table S1).

**Figure 6:**
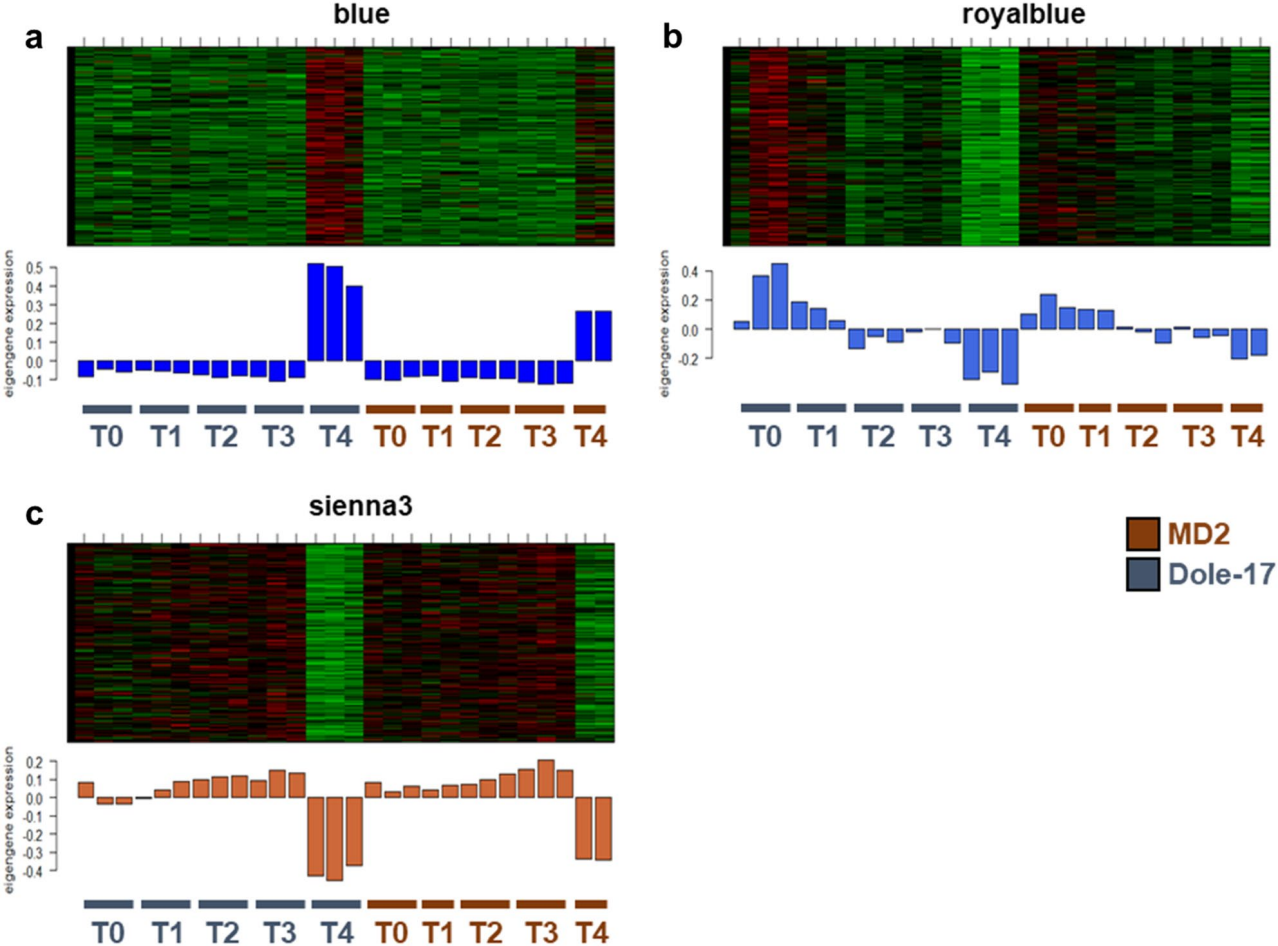
Heatmap and bar plots for (**a**) blue, (**b**) royalblue, and (**c**) sienna3 co-expression modules. Top panels include expression heatmaps for all genes in the module and bottom panels include eigengene expression level bar plots. Eigengene expression (y-axis on bar plots) refers to the representative gene expression profile of a module.

Co-expression modules with strong heatmap signals at the T4 time point were hypothesized to be involved in development of floral organs, therefore, they were selected for further analysis. Modules associated with the T4 timepoint included blue (5,541 genes), royalblue (560 genes), and sienna3 (1,609 genes) (Table 2; Fig. 6a–c). The blue module represented up-regulated genes, while royalblue and sienna3 modules represented down-regulated genes at the T4 time point.

Functional annotation of genes in the blue module revealed that they likely play an important role in nutrient biosynthesis that contributes to flower and seed development. For example, enriched GO terms in the blue module included “GO:0005975 carbohydrate metabolic process” and “GO:0015979 photosynthesis”. The expression levels and functional annotation results for the blue module indicated that this module is likely involved in and important for flower development.

The royalblue and sienna3 modules both displayed down-regulation of gene expression in the T4 time point (Fig. 6b,c), therefore, these modules could provide some insight about what genes are down-regulated after floral induction has already occurred. Functional annotation results indicated that the genes clustered into the royalblue module function in responding to environmental stimuli and hormonal signaling. For example, GO terms that were enriched in the sienna3 module included “GO:0009755 hormone-mediated signaling pathway” and “GO:0040029 epigenetic regulation of gene expression”, two biological processes necessary for vegetative-to-floral meristem transition^30,36,79^.

A large portion (70%) of the core flowering genes identified by differential expression analysis were within one of the three T4-related co-expression modules. A total of 262, 20, and 39 core flowering genes were in the blue, royalblue, and sienna3 modules, respectively (Supplementary Figure S5a). All 262 core flowering genes in the blue module were up-regulated, while all 20 in the royalblue, and all 39 in the sienna3 modules were down-regulated in the T4 time point. To narrow down what the potential functions of these co-expression modules in flower development, the overlapping genes between core flowering genes and hub genes from the blue, royalblue, and sienna3 modules (Supplementary Figure S5b) were further investigated.

The blue module contained 24 hub genes that were also core flowering genes, including genes encoding an acetylornithine deacetylase-like enzyme, five chlorophyll-binding proteins involved in photosynthesis, carotenoid and anthocyanin biosynthesis pathway proteins involved in petal pigmentation, an *AP2/ERF* TF in the ethylene-activated signaling pathway, and multiple transmembrane transporter proteins. Expression patterns, number of core flowering genes, and predicted function provided strong evidence that the blue module represents a subset of genes important in floral organ genesis. Acetylornithine deacetylase functions in maintaining optimal nitrogen:carbon (N:C) ratio during flowering in *Arabidopsis* and affects plant sensitivity to glucose^81^. These results also agreed with previous studies which have reported that transporter activity oscillates in correlation with photosynthetic activity in pineapple^82^. Starch accumulation is a necessary process for pineapple flowering, and the prevention of starch accumulation in pineapple leaves has been experimentally shown to prevent natural flowering^6^. Sucrose accumulation could be attributed to early development of fruit tissue during the T4 time point^52,83^. Based on the gene expression and functional annotation results for the blue module, we can determine that this module represents genes involved in biosynthesis of carbohydrates that are needed by the plant for developing reproductive tissues.

The royalblue module contained only 1 hub gene that was also core flowering gene, which was an ABA receptor (*PYL12-like*). According to previous research, PYL proteins function in abiotic stress response^12,79,84,85^, therefore, this gene was likely involved in ABA-mediated stress responses prior to flowering induction. The sienna3 module contained 3 hub genes that were also core flowering genes, a B3-domain-containing TF (*DLN34*), a C2H2-type zinc finger protein (*ZFP4*), and a homogentisate phytyltransferase protein (*HPT1*). *DLN34* functions as a transcriptional repressor to regulate a range of developmental processes^86^. *ZFP4* functions as a negative regulator of glucosinolate biosynthesis^87^, a type of plant secondary metabolite that functions in defense against biotic stresses^87–89^. Finally, *HPT1* catalyzes one of the steps in the tocopherol (aka vitamin E) biosynthesis^90^, which functions in quenching ROS and accumulates in seeds^90,91^. Genes identified in the royalblue and sienna3 modules indicated that these two modules are likely involved in stress responses and flowering regulation.

When considering the gene expression patterns of the blue, royalblue, and sienna3 modules, the functional annotation results for these modules agreed with what processes we would expect to see during the process of stress-induced flowering in pineapple. Genes related to stress response and regulation of flowering time were more highly expressed in T0 through T3 samples, while genes related to nutrient accumulation were more highly expressed in the T4 time point during the metabolically costly processes of inflorescence, flower, and fruit formation.

## Discussion

Cool night temperatures and natural cold fronts in tropical growing regions are associated with untimely natural induction of flowering in pineapple. However, few studies have attempted to identify the genetic mechanisms connecting cold stress response and flowering time for this important fruit crop. In this study, clear differences in cold stress response were observed between a newly developed late-flowering genotype, Dole-17, and a widely used commercial variety that is susceptible to precocious flowering, MD2. These two genotypes also displayed a noticeable phenotypic difference in flowering time. *Arabidopsis* and rice genome annotations were used to identify potential flowering orthologs in pineapple. These results were combined with cold-responsive genes identified by gene expression analysis to determine which genes are potentially involved in cold-mediated flowering or vernalization. We hypothesized that the phenotypic differences observed between the two genotypes were likely due to differences in stress responses that triggered genes that regulate flowering time.

In this study, we identified DEGs and co-expressed gene modules in an effort to elucidate the genetic mechanisms driving cold stress responses in pineapple. Functional annotation of the DEGs between genotypes (e.g., Dole-17_T0_ vs MD2_T0_, etc.) determined they are involved in stress response and plant defense. Given the majority of DEGs (85%) from the comparisons between genotypes for each time point (T0-T3) are down-regulated in Dole-17, these results indicate that MD2 may have a more dramatic transcriptional response to cold stress compared to Dole-17. Gene expression levels for eleven of the stress- and flowering-related DEGs identified here were validated by RT-qPCR analysis. These results confirmed that Dole-17 and MD2 had significant differences in expression of genes that likely trigger flowering in pineapple, such as ethylene-responsive TFs and flowering time controlling proteins.

This study supports that the Dole-17 and MD2 pineapple genotypes have a difference in their tolerance to low temperatures, as shown by the differences in transcriptional responses associated with physiological changes for adapting to stress, such as alteration of wax biosynthesis, anthocyanin production, and stomatal movement^12,47^. One of the most noticeable transcriptional changes upon cold treatment included changes in expression of regulatory genes, particularly TFs that are temperature-stress related, ABA-responsive, and/or ethylene-responsive. Some of the differentially expressed TF families included *AP2/ERF, bHLH, HSF, MADS*, and *FAR*, which have been previously implicated in playing a role in stress tolerance and flowering time in pineapple and other species^17,24,29,47,74,92–96^. Our results demonstrated the ethylene pathway plays a large role in cold response in pineapple, as previously seen in cultivar ‘Shenwan’^12^. The cold-induced flowering mechanism for pineapple genotypes MD2 and Dole-17 were compared to each other while also using two well-studied species (i.e., *Arabidopsis* and rice) as references for understanding potential functions of candidate genes. In our study, Dole-17 exhibited an increase in cuticular wax production^48,49^ and anthocyanin biosynthesis^18,46^, which may have contributed to delaying flowering onset while MD2 appeared to have greater susceptibility to cold stress. As a result of the MD2 genotype being unable to mitigate cold stress, our results suggested that the increased stress endured by MD2 versus Dole-17 lead to increased ethylene production, signaling, and possibly sensitivity, ultimately leading to earlier flowering induction. Our results share similarities with previous studies in other plant species with precocious flowering, such as canola^37^ and bamboo^32,97^, which suggest that these species always have the potential to flower, but sensitivity of plants to environmental cues heavily affects flowering time. In pineapple, it is likely that these environmental cues are needed to stimulate ethylene production, leading to flowering^7,12,15,35^.

Overall, gene expression results suggested that the vernalization pathway is naturally triggered in pineapple by periodic cold events in tropical growing regions, however, Dole-17 and MD2 genotypes display differing levels of expression of vernalization pathway, as well as genes in other flowering-related pathways, including photoperiod, GA, and autonomous. Some of the differentially expressed cold-responsive genes were also involved in the transition from vegetative to floral meristem, such as *VRN5*^35^, *CLF*^38^, *YABBY4*^98^, and several serine/threonine-protein phosphatases^99–101^. These results suggest that in addition to ethylene pathway genes, meristem identity genes are also potential candidates for identification of cold tolerant genotypes of pineapple. In addition, this report also identifies a core set of genes involved in developing floral organs and other reproductive tissues.

In summary, our analysis shows for the first time the role of different physiological adaptations, metabolite production, and ethylene-mediated responses that contribute to cold tolerance in pineapple.

## Materials and methods

### Plant materials and experimental design

Two pineapple varieties were used to perform a comparative transcriptome analysis for cold stress response, a precocious flowering-susceptible commercial variety (MD2) and a newly developed tolerant hybrid accession (Dole-17). Mature, vegetative-stage pineapple plants of both genotypes were grown at the Dole plantation field in La Ceiba, Honduras arranged in a randomly complete block design, and subjected to natural cold events in December 2020. Fresh meristem tissue (1 cm radius × 1 cm deep piece of very center of stem) from field-grown Dole-17 and MD2 pineapple plants were harvested at five time points (T0-T4) and placed immediately on dry ice. Time point T0 represents the vegetative stage before treatment or visible flowering induction occurs (i.e., baseline). Time points T1, T2, and T3 represent three post-cold event collection stages. Here, a cold event is defined as being ≤ 18 °C for at least 8 h. The three cold events in this study were spaced at least 1 week apart to ensure that any changes in gene expression were due to each distinct cold event and not a result of enduring effects from a previous event. Samples were collected between 18-24 h after each cold event subsided. Time point T4 represents the first visible stage of flowering (also known as button stage), therefore the tissue collected at T4 was the floral meristem. Three biological replicates were harvested for each sample. Experimental research and field studies on plants, including the collection of plant material, complied with relevant institutional, national, and international guidelines and legislation.

### RNA extraction and sequencing

Total RNA was extracted from collected meristem tissues using the Qiagen RNeasy plant mini kit (Qiagen, Hilden, Germany). RNA integrity was evaluated on a 1.0% agarose gel, quantified using Qubit 2.0 fluorometer (Invitrogen, Waltham, MA), and purity tested using NanoDrop (Fisher Scientific, Waltham, MA). Illumina libraries were prepared using the NEBNext Ultra Directional RNA Library Prep Kit for Illumina (New England Biolabs, Ipswich, MA) and sequenced at the NCSU Core Genomic Sciences Laboratory (NCSU-GSL) on the Illumina NovaSeq 6000. The RNA-seq reads were quality-checked with FastQC (https://www.bioinformatics.babraham.ac.uk/projects/fastqc/) and cleaned using Trimmomatic^102^ (parameters:ILLUMINACLIP:2:30:10:2:TRUE SLIDINGWINDOW:10:30 LEADING:5 TRAILING:5 HEADCROP:10 MINLEN:75), which removed low-quality and adapter sequences. The cleaned sequences were then retained for expression analysis.

### Differential expression analysis

Pairwise differential expression analysis was performed with RNA-seq data from Dole-17 and MD2 meristem tissue. This analysis was performed using RSEM v.1.3.3 and the –bowtie2 parameter^103^ for alignment and transcript quantification; the CDS sequences of the pineapple MD2 v2 predicted genes were used as a reference for the alignment^11^. Differentially expressed genes (DEGs) were then identified using DESeq2 v.3.15 (with default parameters)^104^ with the raw count data from RSEM as input.

DEG analysis was performed at 3 levels: first, the T0 time point was used as a pre-cold event reference point for comparison against post-cold event time points (e.g., T0 vs T1, T0 vs T2, and T0 vs T3) to reveal genes that play a role in stress response in pineapple; second, step-wise comparisons for T1, T2, and T3 time points (e.g., T1 vs T2, T2 vs T3) were used to identify genes whose expression was altered by repeated exposure to cold temperatures; finally, comparisons within time point (T0, T1, T2, T3) and between genotypes were used to reveal genes with significantly different expression patterns between MD2 and Dole-17, providing insight into differences in cold stress response between the two genotypes. Functional annotation of DEG subsets was used to further investigate the genetic mechanism that can trigger flowering initiation in response to cold stress. In addition, DEG analysis for the T4 time point versus T0, T1, T2, and T3 time points for each genotype was performed to identify a core set of genes up- or down-regulated during flower development in pineapple.

### Gene co-expression analysis

Co-expression networks were constructed using the WGCNA v.1.70-3 package in R studio v.2021.09.2^105^. The entire set of predicted genes for pineapple MD2 v2 were used for the co-expression network analysis with TMM-normalized count data for all genes (see Differential expression analysis section) as the input. The parameters used for module construction with WGCNA were as follows: step-by-step network construction and module detection, hierarchical clustering method = average, signed network, power = 12; minModuleSize = 30. WGCNA was run twice, once with and once without the T4 samples. The first run included the T4 samples, and allowed us to identify modules associated with floral development. The second run excluded the T4 samples in order for us to gain better resolution for identifying modules associated with stress responses that led to floral induction and differences in flowering time between MD2 and Dole-17.

After clustering and removal of outlier samples (MD2_T1_ rep2 and MD2_T4_ rep3), modules were constructed using the step-by-step network construction approach. To reduce the number of network modules to a reasonable number that could be correlated with time point, genotype, or condition (timepoint x genotype), smaller modules (number of genes < 30) with highly similar expression profiles were merged (MEDissThres = 0.25). Modules that were associated with time point, genotype, or condition were identified based on module-trait correlation (R^2^ ≥ 0.5) calculated by WGCNA and used for further analyses. Hub genes for selected modules were identified by filtering genes in modules with high significance for condition (GS ≥|0.5|) as well as high module membership (MM ≥ 0.9).

### Identification of orthologous flowering genes in pineapple

Flowering genes in pineapple were identified via orthology with *Arabidopsis* and rice. These 2 species have well-annotated genomes that have high numbers of experimentally validated gene functions. In this study, the *A. comosus* MD2 v2 gene prediction was used for identification of candidate flowering genes for pineapple^11^. Genes for ortholog prediction were obtained for the *Arabidopsis* Araport 11 and rice IRGSP-1.0 genomes from the TAIR (https://www.arabidopsis.org, visited on 01/14/2022) and RAP-DB (https://rapdb.dna.affrc.go.jp/, visited on 01/14/2022) databases, respectively. The flowering genes for rice were obtained from 2 publications about genetic mechanisms regulating flowering time in rice^30,69^. The list of flowering genes in *Arabidopsis* was obtained by typing the key word “flower” into the TAIR database search function. These two lists of flowering genes were used to filter orthologous pairing results and identify candidate flowering genes in pineapple. Ortholog prediction for pineapple, rice, and *Arabidopsis* was performed using OrthoMCL v.2.0.9 (https://orthomcl.org).

Two-hundred and nineteen flowering-related genes were identified in pineapple MD2 v2 based on orthology with *Arabidopsis* (161 orthologs) and rice (96 orthologs). To validate the results of the orthologous analysis, pineapple genes were used for gene enrichment analysis. Functional annotation of the orthologous genes revealed they play important roles in light and abiotic stress response, epigenetic regulation of gene expression, the transition from vegetative to floral meristem, and floral organ development. Indeed, enrichment analysis for GO terms and network pathway analysis results supported that these genes function in flowering. Biological process GO terms that were over-represented in this gene set include regulation of flower development, vernalization response, and genomic imprinting. Plant reactome pathways with the most pineapple flowering orthologs included Brassinosteroid signaling, Reproductive meristem phase change, and Abscisic acid (ABA) mediated signaling. KEGG pathways with the most pineapple flowering orthologs included Circadian rhythm–plant, Lysine degradation, and Spliceosome. Overall, the results confirmed that this subset of genes were truly flowering related genes in pineapple. This subset of genes was then used for subsequent analysis, such as identifying the overlap between these genes and identified DEGs and co-expression modules.

### Functional enrichment

Functional annotation data, including BLAST homology, gene ontology (GO), protein domains, and regulatory sequences were obtained for the MD2 v2 predicted genes^11^. Selected subsets of genes were subjected to enrichment analysis using the Fisher’s Exact Test option in Omicsbox v.2.1.14^106^ with the following parameters: FDR-corrected *p*-value < 0.05, two-tailed, GO IDs, GO categories Biological Process, Molecular Function, and Cellular Component. For all tests performed, the entire set of functionally annotated MD2 v2 genes was used as the reference set.

### Biological pathway and interactome analysis

A protein interaction network was built to identify functional associations for protein-encoding genes potentially involved in cold stress response. For this analysis, DEGs between Dole-17 and MD2 genotypes were identified and filtered for genes that were DE between at least 2 cold event time points (T1, T2, and T3). *A. thaliana* orthologs of DEGS were obtained with TAIR protein database using blastx tool (e-value = 1e − 10; % sequence identity and query sequence coverage > 60%). The interaction network of cold stress-associated genes was created by mapping the orthologs with the *A*. *thaliana* STRING interactome database and analyzed using Cytoscape v.3.9.1 and the stringApp v.2.0.1 application in Cytoscape^107,108^. Functional modules in the constructed network were identified using the MCODE tool, which were subjected to enrichment analysis^109^. Pearson’s correlation was computed based on the differential expression profile of nodes in the predicted network to identify the co-expression between the significantly enriched pathways (*p*-value ≤ 0.05).

### Real-time quantitative (RT-qPCR) validation

RT-qPCR was used to test the expression of 11 DEG genes. Primers were designed using NCBI Primer-Blast (https://www.ncbi.nlm.nih.gov/tools/primer-blast/) and sequences are reported in Supplementary Table S5. Isolation and QC of total RNA for MD2 and Dole-17 are described previously in this paper (see RNA extraction and sequencing section). For RT-qPCR validation, RNA (~ 2 µg) was synthesized into cDNA by using the SuperScript III First-Strand Synthesis System (Invitrogen, USA) through a one-step method. RT-qPCR was performed on a LightCycler 480 II (Roche, Switzerland) using SYBR Green qPCR Master Mix (Thermo Fisher Scientific, USA) with three biological and technical replicates for each gene. Expression levels of the tested genes were normalized to the transcript levels of the internal reference pineapple β-Actin gene^110,111^. The relative expression levels of genes were calculated using the 2^(− ΔΔCt) method with MD2_T0_ as the reference sample. The GraphPad Prism 9 (Dotmatics, USA) software was used for conducting statistical analyses (significance *P* < 0.05) (GraphPad Software Inc.).

### Ethics approval and consent to participate

The samples that were collected for this study were collected with the permission of Dole Food Company. These samples originated from a commercially cultivated species (*Ananas comosus* var. *comosus*), not a species at risk of extinction or endangerment. In addition, no seed or propagative material was collected for this research.

### Supplementary Information

Supplementary Tables.

Supplementary Figures.

## Data Availability

Data including raw sequencing reads have been deposited in the NCBI under BioProject ID PRJNA966704 and submission ID SUB13172003. Reviewer link: https://dataview.ncbi.nlm.nih.gov/object/PRJNA966704?reviewer=p0ep2eo9fvcqc5hnps4hfpoq59.
