# Supplementary material for: Comparative transcriptome analysis reveals candidate genes for cold stress response and early flowering in pineapple
Source: Sci Rep. 2023 Nov 2;13:18890. doi: 10.1038/s41598-023-45722-y (PMC10622448; doi:10.1038/s41598-023-45722-y)
Supplement: Supplementary file 2 — Supplementary Figures. [file 41598_2023_45722_MOESM2_ESM.docx]

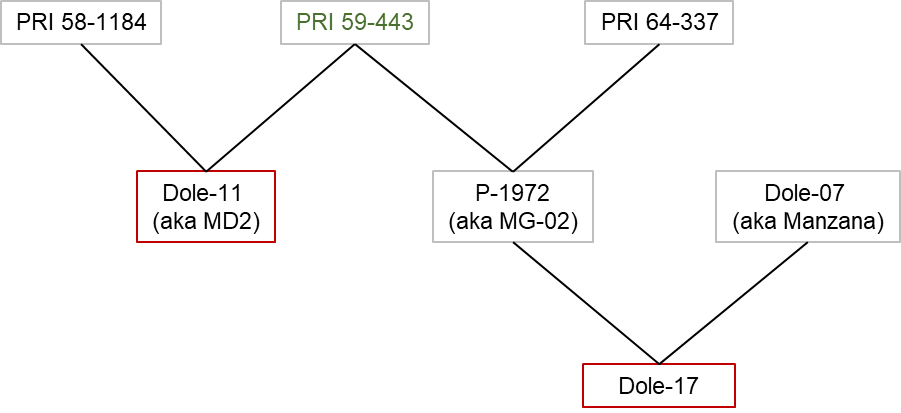


**Supplementary figure S1.** Pedigree chart showing the relationship between Dole-17 and MD2 genotypes.


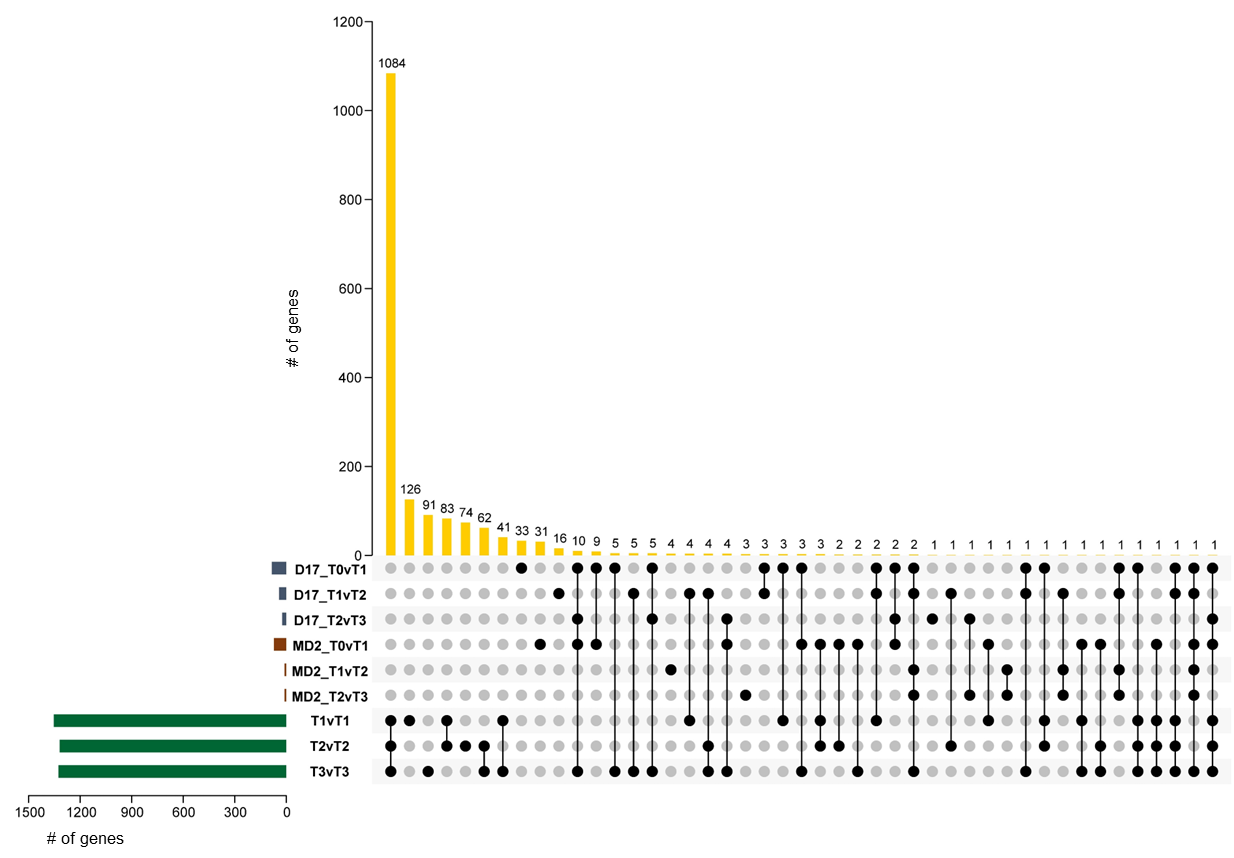


**Supplementary figure S2.** Upset plot showing overlap of DEG sets (T0 through T3 samples only).


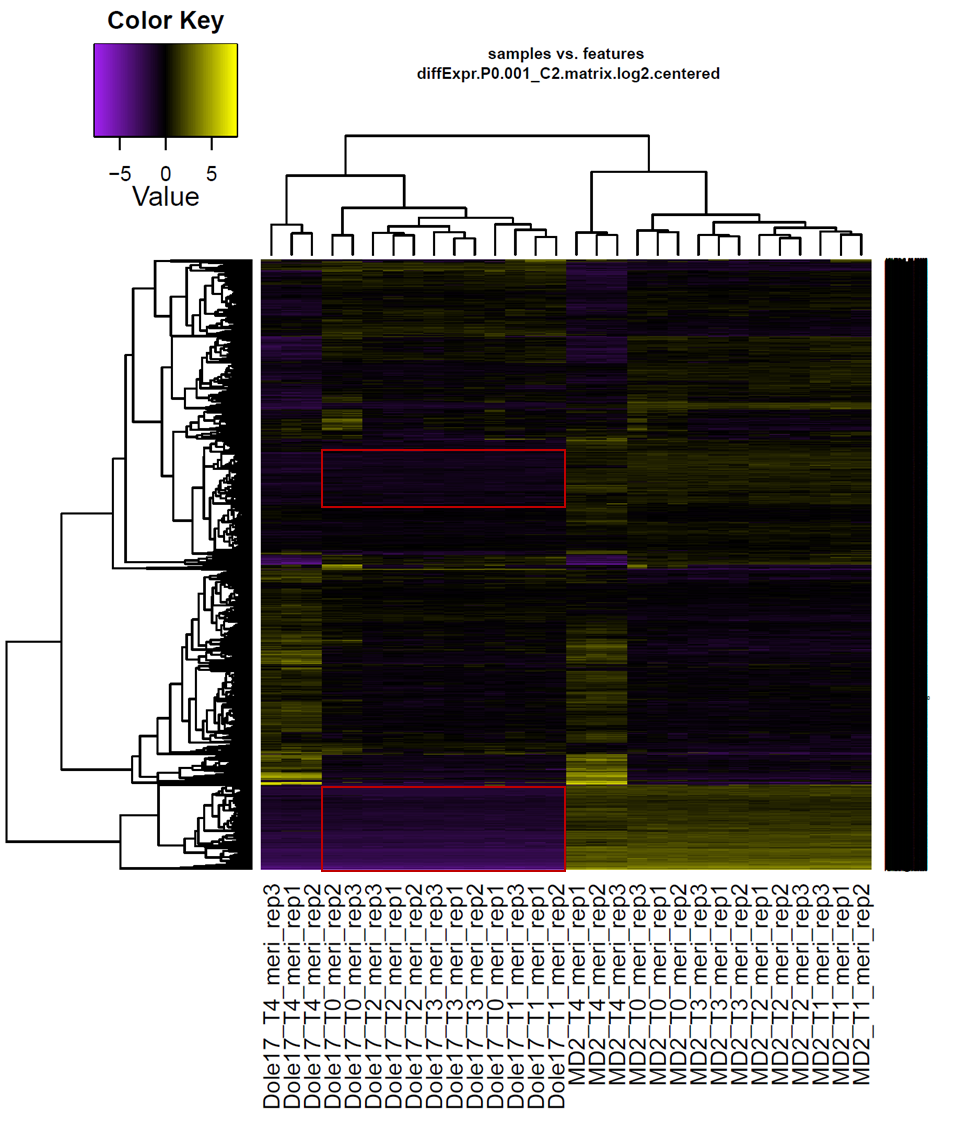


**Supplementary figure S3.** Heatmap plot of normalized expression levels across all samples used in this study. Red boxes indicate genes consistently down-regulated in Dole-17 relative to MD2.


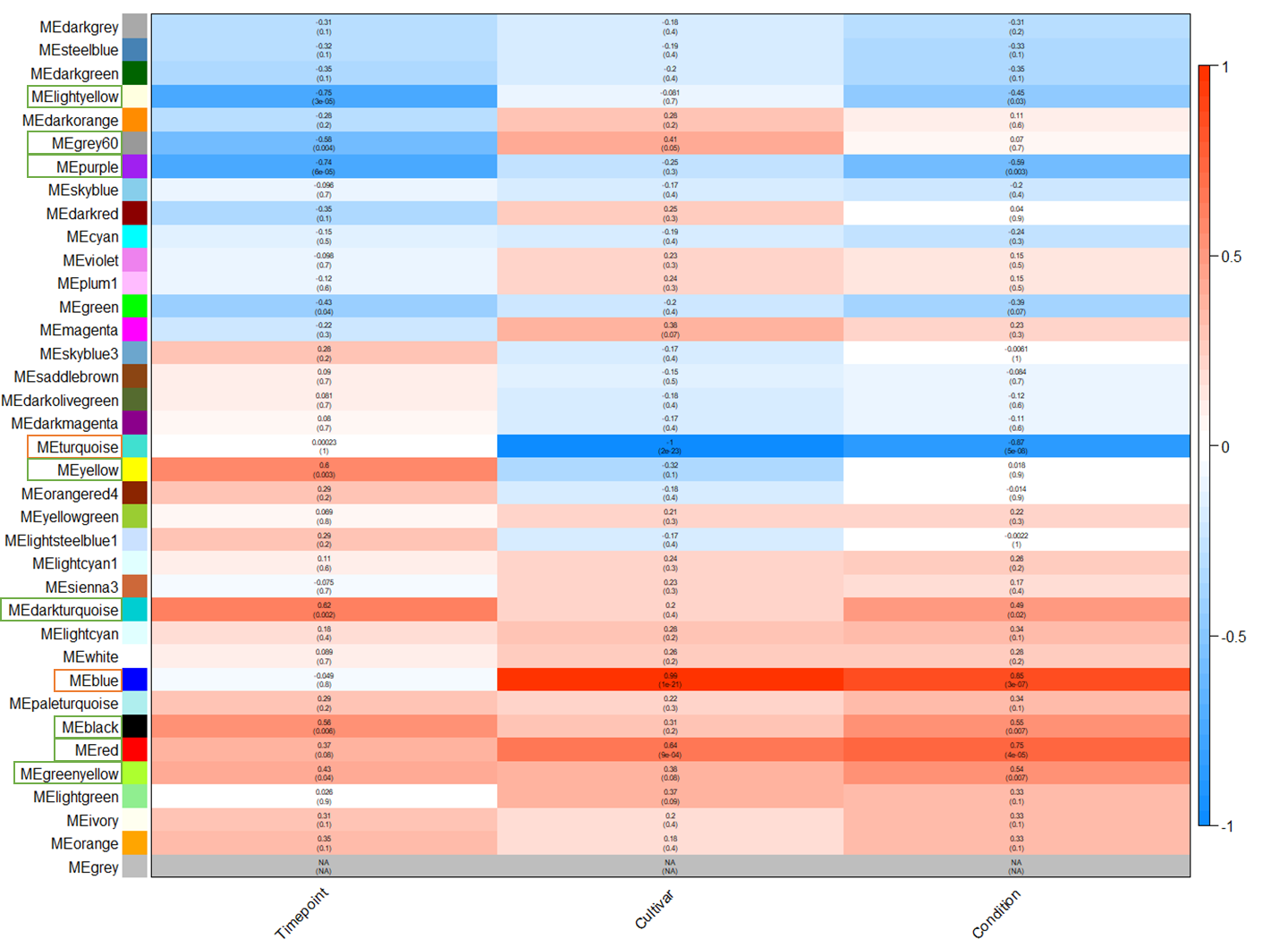


**Supplementary figure S4.** Correlation heatmap for co-expression modules identified in T0 through T3 samples. Green boxes indicate modules with eigengene expression patterns associated with stress and orange boxes indicate modules with genotype-specific eigengene expression patterns.


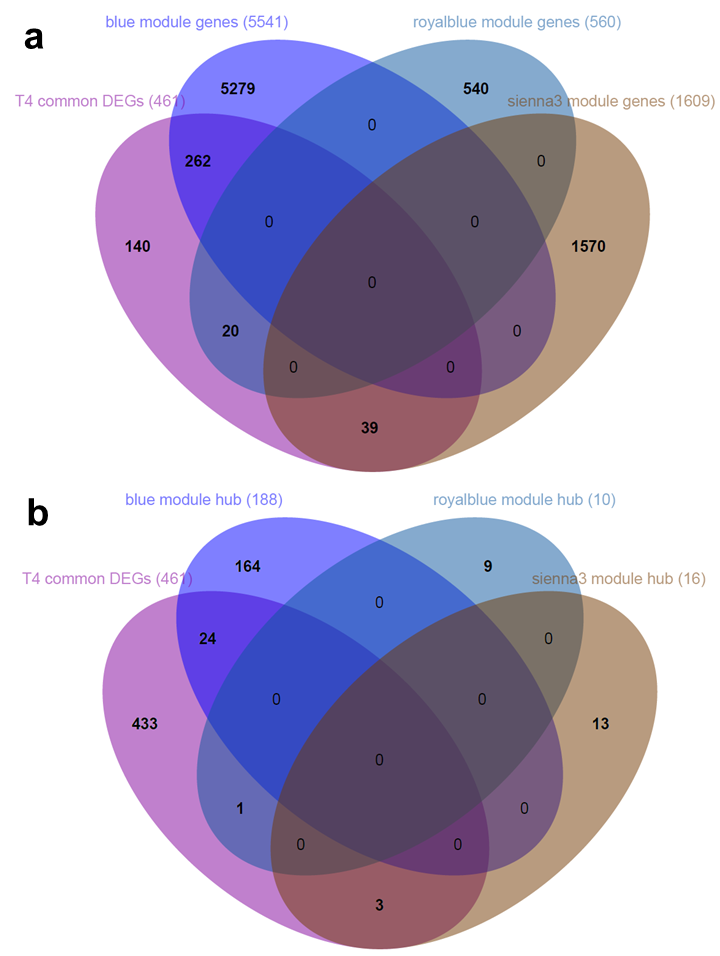


**Supplementary figure S5**. Venn diagrams depicting overlap between core flowering genes identified in this study and co-expression modules associated with time point T4 (blue, royalblue, and sienna3). Time point T4 was collected during the earliest visible stage of flowering. Panel a) represents overlap between core flowering genes and all genes in the modules shown; b) represents overlap between core flowering genes and hub genes for the same modules. Hub genes are considered genes within a module with high connectivity (see materials and methods).
